# Vascular Dysfunction in Diabetes and Hypertension: Insights into HSP70

**DOI:** 10.3390/cells15181652

**Published:** 2026-09-13

**Authors:** Swasti Rastogi, Jessica Liaw, Yair Lara-Blanco, Valentina Ochoa Mendoza, Kenia Pedrosa Nunes

**Affiliations:** Laboratory of Vascular Biology, Department of Biomedical Engineering and Science, Florida Institute of Technology, Melbourne, FL 32901, USA

**Keywords:** vascular dysfunction, diabetes, hypertension, HSP70

## Abstract

**Highlights:**

**What are the main findings?**

- HSP70 is differentially regulated in diabetes and hypertension, with its effects context-dependent and varying by cellular localization, tissue, sex, and disease stage.
- HSP70 is associated with Ca^2+^ handling and vascular function, and evidence supports its involvement in diabetes-associated vascular dysfunction.

**What are the implications of the main findings?**

- HSP70 cannot yet be considered a universal biomarker for vascular stress or CVDs but should be interpreted in the context of the disease and tissue studied.
- Establishing whether HSP70 acts as a causal mediator, biomarker, or adaptive response is important before it can be targeted therapeutically.

**Abstract:**

Diabetes and hypertension are closely interlinked and among the most prevalent diseases that significantly increase cardiovascular risks. They can be caused by a wide range of factors, which often affect overlapping molecular pathways, resulting in vascular dysfunction. Hyperglycemia in diabetes and increased angiotensin II levels in hypertension act similarly to promote vascular dysfunction by triggering pathways leading to oxidative stress, low-grade inflammation, and vessel remodeling. In this context, Heat Shock Protein 70 (HSP70), a ubiquitous molecular chaperone that exists locally (iHSP70) and systemically (eHSP70) and has opposing biological effects, is emerging as a new component associated with vascular dysfunction in chronic conditions. While elevated levels of eHSP70 in diabetes and hypertension have been associated with low-grade inflammation, iHSP70 appears to regulate intracellular calcium handling—a hallmark mechanism disrupted in both pathologies. This review focuses on the role of HSP70 as an evolving component of the intricate mechanisms underlying vascular dysfunction in these two pervasive diseases, which are very likely to coexist and represent a growing burden worldwide, while addressing whether HSP70 actively contributes to the vascular dysfunction or reflects an adaptive response to disease-associated stress across tissues and experimental models. Finally, we discuss emerging evidence on the pharmacological modulation of HSP70 and the challenges remaining in defining its role as a biomarker and therapeutic target.

## 1. Introduction

Vascular smooth muscle cells (VSMCs) are major determinants of vessel diameter, wall tension, and vascular tone through their capacity to contract and relax [1,2]. In resistance arteries, such as small arteries and arterioles, VSMCs primarily regulate vessel tone and tissue perfusion, whereas in large elastic and muscular arteries, VSMCs contribute to vessel wall mechanics, compliance, and structural integrity [3]. Although endothelial dysfunction has historically been regarded as the initiating event of vascular dysfunction in cardiovascular diseases (CVDs), much evidence highlights the critical roles of VSMC contractile behavior and phenotypic plasticity. VSMC hypercontractility and phenotypic changes impair vascular reactivity and promote arterial remodeling and stiffening [2]. During vascular injury or disease, VSMCs can shift toward a synthetic phenotype, which often contributes to increased extracellular matrix (ECM) production and alters the vessel’s biomechanical properties [4].

The functional coupling between macro- and microcirculation is increasingly recognized as a key determinant of vascular health. Stiffening of large arteries raises systolic and pulse pressures, allowing greater transmission of pulsatile energy into microcirculation [5]. In turn, structural changes within microcirculation, including arteriolar narrowing and an increased wall-to-lumen ratio, elevate systemic vascular resistance and add further burden on the heart and proximal vessels [6].

Hypertension and diabetes are two of the most prevalent and interrelated cardiometabolic disorders, affecting the vasculature. Together, they drive a substantial proportion of global morbidity and mortality. Despite advances in the management of hypertension and diabetes, vascular dysfunction and end-organ damage remain important clinical concerns, underscoring the need for therapeutic strategies that mitigate the associated vascular damage [7,8,9,10]. While diabetes and hypertension develop differently, they frequently converge on pathways that contribute to vascular dysfunction, including oxidative stress, inflammation, ECM remodeling, and impaired nitric oxide (NO) bioavailability [9,11]. In diabetes, chronic hyperglycemia promotes the formation of advanced glycation end-products (AGEs) and activates pro-inflammatory signaling cascades [12]. In contrast, hypertension exposes the vascular wall to increased mechanical stress and Angiotensin II (Ang II) mediated signaling [13]. Together, these insults promote structural remodeling and blunted vasoreactivity, thereby increasing cardiovascular risk.

In this context, heat shock protein 70 (HSP70), a chaperone with multiple biological functions, has emerged as a potential regulator of vascular function [14]. HSP70 is best known for mediating proteostasis by assisting in protein folding, preventing aggregation, and facilitating the degradation of damaged proteins [15]. Beyond its classical role, HSP70 supports vascular contraction and calcium (Ca^2+^) signaling under physiological conditions [14]. In diabetes, it is hypothesized to act as an inflammatory marker [16], and its blockade is suggested to improve vascular function [17], yet the specific mechanisms remain unclear. In hypertension, HSP70 has been implicated in immune and inflammatory responses [18], but its direct role in hypertensive vasculature remains largely unknown.

Importantly, HSP70 is ubiquitously expressed in cells and can be released to the extracellular milieu in response to physiological stress [19]. In diabetes and hypertension, increased extracellular HSP70 (eHSP70) is often pro-inflammatory, whereas intracellular HSP70 (iHSP70) appears context-dependent [20,21,22,23]. Yet, that dual role depends on its cellular localization, disease state, and surrounding stress environment. However, it remains unclear whether HSP70 contributes to vascular dysfunction or reflects an adaptive response to disease-induced vascular stress and how these effects vary across disease, tissues, and experimental models. Accordingly, this review focuses on HSP70 in vascular dysfunction specifically associated with diabetes and hypertension, highlighting its role, with particular attention to current evidence, associated mechanisms, and key questions for future research to advance the field.

## 2. A New Vascular Player: HSP70

**Figure 1:**
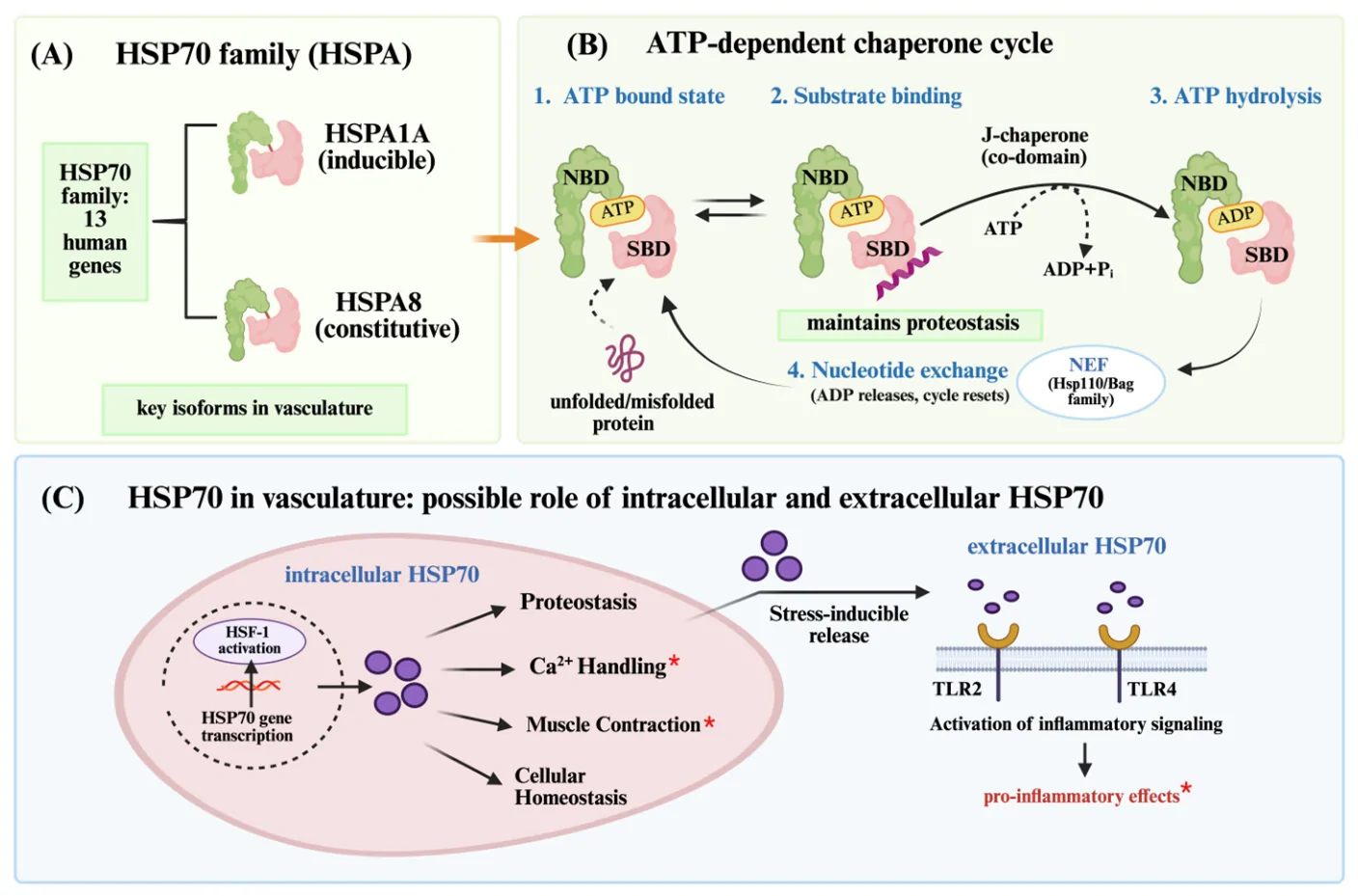
Overview of the HSP70 family, ATP-dependent cycle, and possible roles of intracellular and extracellular HSP70. (**A**) The HSP70 family is encoded by 13 genes in humans, including stress-inducible and constitutive forms, highlighting key isoforms in the vasculature. (**B**) HSP70 functions through an ATP-dependent chaperone cycle. In the ATP-bound state, HSP70 has low substrate affinity. Substrate binding, assisted by J-domain co-chaperones, stimulates ATP hydrolysis, converting HSP70 to the ADP-bound state with higher substrate affinity. Nucleotide exchange factors (NEFs), such as HSP110 and Bag proteins, promote ADP release and ATP rebinding, thereby allowing substrate release and resetting the chaperone cycle. (**C**) Intracellular HSP70 is induced through heat shock factor-1 activation and HSP70 gene transcription. iHSP70 contributes to proteostasis, Ca^2+^ handling, VSMC contraction, and cellular homeostasis. In contrast, eHSP70 can be released under stress and interact with pattern recognition receptors, such as TLR2 and TLR4, thereby promoting inflammatory signaling. *: context-dependent emerging evidence (Created in BioRender. https://BioRender.com/by7v0k3).

HSP70 (HSPA1A in humans, DnaK in prokaryotes) is a highly conserved molecular chaperone critical for proteostasis under physiological and pathological conditions. As a key member of the 13-isoform HSP70 family, it facilitates nascent protein folding, refolding of denatured proteins, membrane translocation, and aggregate disassembly—thereby mitigating proteotoxic stress and preventing apoptosis [15,24]. HSP70 expression is induced by heat shock factors (HSF1), which bind heat shock elements in response to insults (e.g., heat, oxidative damage, hyperglycemia) [25]. The most abundant constitutive (cognate) isoform is HSPA8 (also termed HSC70), which maintains baseline proteostasis in unstressed cells across tissues. Among inducible forms, HSPA1A predominates following stress or agonist stimulation (Figure 1A) [21,26,27]. While the HSP70 family comprises multiple isoforms with varying expression patterns and subcellular localizations, this review collectively uses the term “HSP70’’ to represent the HSP70/HSPA family, unless isoform- and location-specific distinctions are relevant, as summarized in Table 1.

Structurally, HSP70 comprises an N-terminal nucleotide-binding domain (NBD, ~40 kDa) with ATPase activity, a central flexible linker, and a C-terminal substrate-binding domain (SBD, ~25 kDa) that recognizes short hydrophobic peptide motifs on client proteins. The chaperone cycle is allosterically regulated by nucleotide state: in the adenosine triphosphate (ATP)-bound conformation, the NBD and SBD are loosely coupled, allowing rapid substrate binding and release [28]; hydrolysis to adenosine diphosphate (ADP), facilitated by J domain co-chaperones, locks the SBD in a high-affinity state for substrate refolding (Figure 1B). Nucleotide exchange factors (e.g., Hsp110/Bag family) then reset the cycle by promoting ADP release [15].

Beyond its classical role in protein folding, HSP70 is well studied in muscle biology, as it is required for proper contractile function. In skeletal muscle cells, membrane depolarization induces HSP70 expression through a Ca^2+^-dependent signaling mechanism [29]. In cardiac muscle, genetic deletion of inducible HSP70 in mice impairs contractile function and Ca^2+^ handling and is associated with cardiac hypertrophy [30]. Similarly, deletion of HSP70 genes reduces cardiomyocyte shortening and disrupts Ca^2+^ mobilization [30]. Pharmacological inhibition of HSP70 with VER155008 reduces force development in response to an alpha-1 adrenergic agonist [14] and alters intracellular Ca^2+^ levels in isolated aortic rings [31].

HSP70 is broadly expressed across multiple tissues, including the adrenal gland, urinary bladder, breast, bronchus, esophagus, kidney, skin, and prostate [32]. Its expression is cell-, tissue-, organ-, sex-, and/or disease-dependent [21,33,34,35]. For instance, female rats exhibit lower basal aortic HSP70 levels than males and display greater sensitivity to HSP70 inhibition during phenylephrine-induced contraction [33]. However, studies in Sprague–Dawley rats of both sexes suggest that HSP70 expression decreases with age, thereby affecting vascular contractile responses and Ca^2+^ dynamics [34]. For years, HSP70 was thought to reside only intracellularly (iHSP70), but this changed when Asea and Calderwood demonstrated that, in response to stressful stimuli, HSP70 can be exported to the extracellular milieu (eHSP70) and work as a ligand for some membrane receptors such as toll-like receptors 2 and 4 (TLR2 and TLR4) (Figure 1C) [35].

In this review, iHSP70 and eHSP70 refer to intracellular and extracellular localizations, respectively, rather than specific isoforms. Because the biological effects of these two HSP70 pools may vary with context, their relative balance has been proposed as an indirect indicator of the inflammatory state, expressed as the H-index, as suggested by the Chaperone Balance Hypothesis [36]. The H-index is calculated as the eHSP70-to-iHSP70 ratio under disease or experimental conditions (Rj) divided by the corresponding ratio under control conditions (Rc), such that H-index = Rj/Rc. Therefore, the H-index reflects a shift in the HSP70 profile, with higher values being associated with a pro-inflammatory state [36]. However, the H-index has methodological limitations, as it may vary across tissues or biological samples used to quantify HSP70. Consequently, the H-index may not be directly comparable across tissues, experimental models, or diseases. It remains an emerging marker for which a standardized reference has not yet been established, limiting its broader interpretation and clinical application. This becomes especially relevant in diabetes and hypertension, where persistent stress may disrupt HSP70 and its contribution to vascular function, suggesting that HSP70 levels change and imbalances might reflect pathological stress. Concomitantly, this might also indicate the body’s defensive response as it shifts from a physiological to a pathological state.

### The Potential Regulators of HSP70

The expression and activity of HSP70 are dynamically regulated by multiple cellular stressors prominent in vascular disease, including oxidative stress, mitochondrial dysfunction, and inflammation [37,38], which can activate the cellular stress response and modulate HSP70 expression and activity [37]. Importantly, these interactions are likely to be context-dependent, including the compartment in which HSP70 is released, potentially influencing the chaperone’s pro- or anti-inflammatory effects [39].

Oxidative stress caused by increased Reactive Oxygen Species (ROS) has increasingly been associated with the activation of the heat shock response and vascular remodeling [37]. The overproduction of ROS has been implicated in the progression of multiple chronic pathologies, including diabetes and hypertension, while HSP70 may respond to this oxidative environment as part of the cellular defense against oxidative injury [37,40]. Additionally, experimental evidence suggests that HSP70 may also influence antioxidant capacity. For instance, HSP70.1-deficient mice (currently known as the inducible form HSPA1B under the new nomenclature) exhibited reduced Cu/Zn superoxide dismutase protein expression and altered antioxidant enzyme activity, suggesting a possible interaction between HSP70 and endogenous antioxidant defenses [41,42].

Mitochondrial dysfunction may further contribute to HSP70 dysregulation by increasing ROS production, impairing ATP availability, and promoting cellular stress. Since HSP70’s activity is ATP-dependent, disruption of cellular energy metabolism may affect chaperone function and expression, which may be particularly relevant in diabetes and hypertension, where mitochondrial oxidative stress and impaired energy metabolism can coexist with vascular dysfunction [43,44]. In ECs, mitochondria interact with the endoplasmic reticulum (ER) to aid in intracellular Ca^2+^ regulation via mitochondrial Ca^2+^ uptake and cycling, while in VSMCs, mitochondria are positioned in proximity to the muscle-specialized sarcoplasmic reticulum (SR), likely to facilitate mitochondrial–SR coupling required for Ca^2+^ homeostasis and vascular tone modulation [45,46]. Clearly, this organelle plays an important role in Ca^2+^ regulation in the vascular system [38,45]. Additionally, ER stress directly modulates HSP70, as disruption of ER protein-folding homeostasis activates processes closely interconnected with molecular chaperone systems and stress response pathways, although this topic has been more extensively characterized in diabetes [47], than in hypertension [48].

Inflammation is another important regulator, with strong evidence supporting its relationship to HSP70. Pro-inflammatory signaling and disruption of cellular homeostasis can induce iHSP70 expression as part of the cellular stress response, while eHSP70 can interact with pattern-recognition receptors, including TLRs, to modulate downstream signaling [35]. Moreover, increased extracellular release of HSP70 and changes in the eHSP70-to-iHSP70 balance have been implicated in inflammation and metabolic disease [36]. Thus, HSP70 may participate in a bidirectional relationship with inflammatory pathways, although its biological effects appear to depend on localization, concentration, and cellular context, and may be particularly relevant to hypertension- and diabetes-induced cellular stress within the vascular wall [49,50].

Importantly, these pathways should not be considered isolated mechanisms. Oxidative stress can promote mitochondrial and ER dysfunction, while mitochondrial ROS can amplify inflammatory signaling and endothelial dysfunction, particularly under chronic metabolic stress in both diabetes and hypertension [43,51]. For this reason, it is of utmost importance to investigate whether HSP70 primarily represents an adaptive defense mechanism, contributes to inflammatory signaling, or participates in both processes during hypertension- and diabetes-associated vascular dysfunction. HSP70 is increasingly implicated in the pathogenesis of CVD and associated comorbidities [52] and may represent a convergence point within an interconnected stress response network, linking cellular stress responses to changes in vascular function.

## 3. The Dysfunctional Vasculature in Diabetes and Hypertension

**Figure 2:**
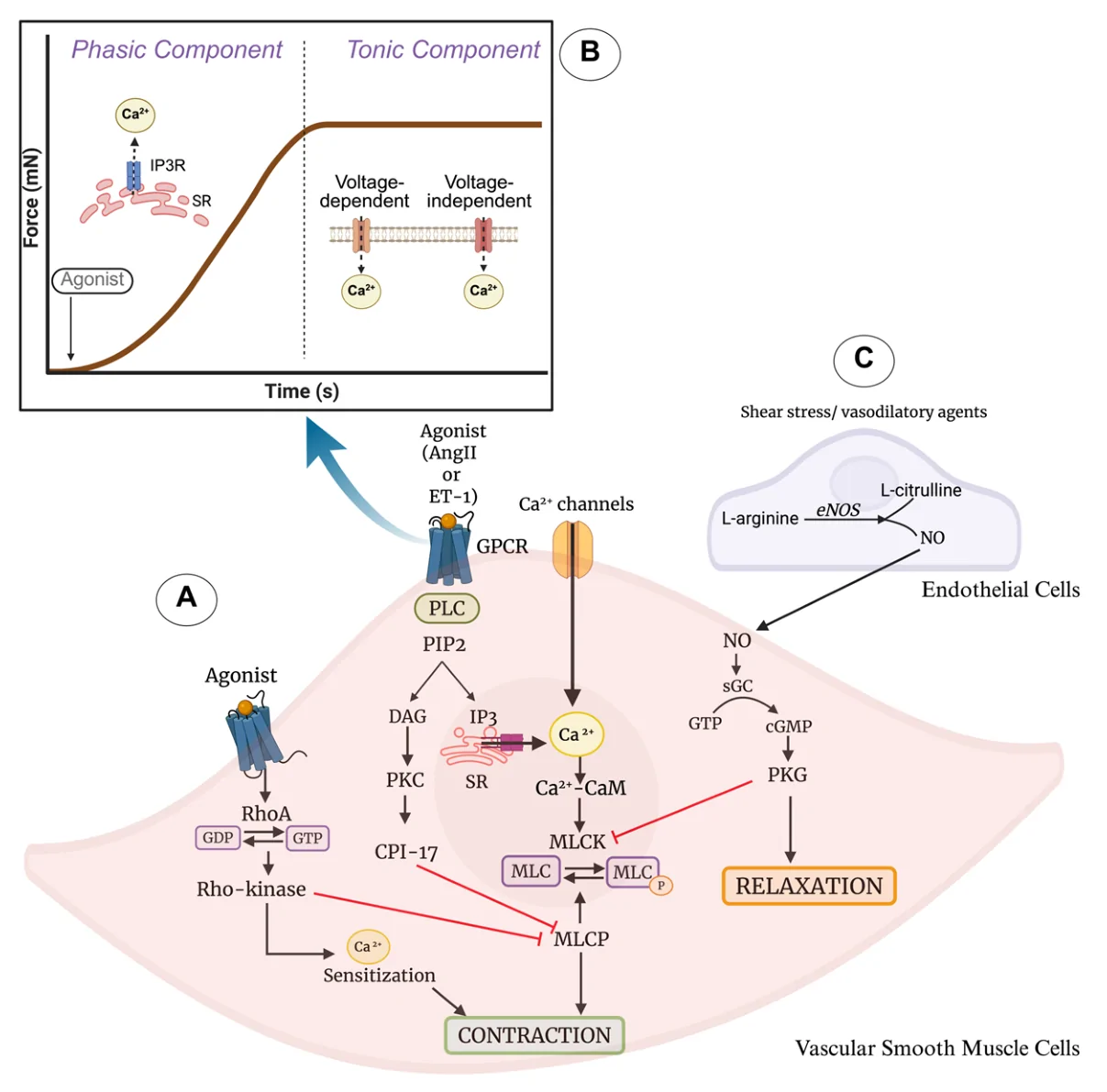
Schematic representation of canonical signaling pathways regulating VSMC contraction and relaxation. (**A**) Vasoactive agonists activate receptor-mediated signaling pathways that increase intracellular Ca^2+^ and promote contraction via phosphorylation of MLC and by parallel pathways, such as RhoA/Rho-kinase and PKC, that enhance Ca^2+^ sensitivity by inhibiting MLCP. (**B**) The panel illustrates the biphasic nature of VSMC contraction. The rapid phasic response or Ca^2+^ efflux is initiated by the IP3R-mediated release of Ca^2+^ from the SR. This is followed by a slower, sustained tonic phase of Ca^2+^ influx, mediated by voltage-dependent and voltage-independent channels. (**C**) In contrast, endothelial-derived NO stimulates cGMP signaling, leading to reduced intracellular Ca^2+^ levels and relaxation. Together, these pathways regulate vascular tone by coordinating contraction and relaxation mechanisms. eNOS: endothelial nitric oxide synthase; NO: nitric oxide; sGC: soluble guanylyl cyclase; GDP: guanosine diphosphate; GTP: guanosine triphosphate; cGMP: cyclic guanosine monophosphate; PKG: protein kinase G; GPCR: G-protein coupled receptors; Ang II: angiotensin II; ET-1: endothelin-1; PLC: phospholipase C; PIP2: phosphatidylinositol 4,5-bisphosphate; DAG: diacylglycerol; IP3: inositol 1,4,5-triphosphate; IP3R: inositol 1,4,5-triphosphate receptors; PKC: protein kinase C; CPI-17: protein kinase C-potentiated phosphate-1 inhibitor of 17 kDa; CaM: calmodulin; MLCK: myosin light chain kinase; MLC: myosin light chain; MLCP: myosin light chain phosphatase; RhoA: ras homolog family member A; Rho-kinase: rho-associated coiled-coil kinase. (Created in BioRender. Nunes, K. (2026) https://BioRender.com/qiof0n6).

In VSMCs, agonist-induced contraction is mediated predominantly by increases in cytosolic Ca^2+^ and changes in Ca^2+^ sensitivity, although contraction can also be initiated by mechanical depolarization and stretching [53,54]. The canonical mechanism leading to contraction is summarized in Figure 2A. Increased Ca^2+^ binds calmodulin (CaM), activating myosin light chain kinase (MLCK), promoting myosin light chain phosphorylation and contraction, while Rho-kinase and protein kinase C (PKC) enhance Ca^2+^ sensitivity and sustain contraction. In response to contractile agonists, such as Phenylephrine (PE), the response to Ca^2+^ surge in VSMC is typically biphasic, as illustrated in Figure 2B: the initial phasic phase is primarily facilitated by Ca^2+^ release from the sarcoplasmic reticulum (SR) through inositol 1,4,5-trisphosphate receptors (IP3Rs), whereas the sustained tonic phase is facilitated by Ca^2+^ influx through plasmalemmal voltage-dependent and -independent Ca^2+^ channels [55].

In contrast, ECs promote vascular relaxation primarily through NO production, the principal endothelium-derived vasodilator that acts on underlying VSMCs [56]. Once produced, NO diffuses into VSMCs and activates soluble guanylyl cyclase (sGC), increasing cyclic guanosine monophosphate (cGMP) and activating protein kinase G (PKG), which promotes vasorelaxation by decreasing intracellular Ca^2+^ and myofilament Ca^2+^ sensitivity, as illustrated in Figure 2C [56,57]. Normally, these pathways work in balance, whilst maintaining vascular tone. However, under pathological conditions, disruption of this coordination contributes to vascular dysfunction.

Diabetes and hypertension are major drivers of vascular dysfunction and share several interconnected mechanisms despite arising from distinct metabolic and hemodynamic disturbances. In diabetes, chronic hyperglycemia promotes excessive production of ROS, the activation of protein kinase C (PKC) and the polyol pathway, and the formation of AGEs, collectively impairing NO signaling and endothelium-dependent vasodilation, while also altering VSMC contractility [51,58,59,60]. Concomitantly, pro-inflammatory mediators such as tumor necrosis factor alpha (TNF-α), interleukin-6 (IL-6), and MCP-1 further potentiate vascular inflammation, promoting leukocyte adhesion, VSMC proliferation, and ECM remodeling [61]. These alterations culminate in vascular dysfunction—a key event linking metabolic dysregulation to the progression of diabetic complications [61]. Although these vascular consequences are common in both major forms of diabetes, the initiating pathophysiological mechanisms differ.

In type 1 diabetes (T1D), autoimmune-mediated destruction of pancreatic β-cells results in absolute insulin deficiency and consequent hyperglycemia [62]. In contrast, T2D is characterized by impaired insulin signaling, which contributes to endothelial dysfunction, in part by reducing phosphatidylinositol 3-kinase/protein kinase B (PI3K/Akt)-dependent eNOS activation, while vasoconstrictor and pro-remodeling pathways remain relatively preserved or enhanced [63,64]. Altered insulin and growth factor signaling within VSMCs further promotes a hypercontractile and pro-remodeling phenotype, amplifying these endothelial defects at the level of the vessel wall [59].

Experimental and clinical studies in diabetes have shown enhanced myofilament Ca^2+^ sensitivity in diabetic vascular beds, particularly in muscular arteries, contributing to increased basal tone and exaggerated vasoconstrictor responses [65]. This enhanced Ca^2+^ sensitivity is associated with increased activity of RhoA/Rho-kinase and PKC signaling, which promote sustained MLC phosphorylation and contraction [66,67,68]. In addition to these alterations in contractile signaling, VSMCs can undergo phenotypic switching from a differentiated contractile behavior toward a synthetic one characterized by increased proliferation, migration, and ECM synthesis [69,70]. These changes contribute to medial thickening, fibrosis, arterial stiffening, and impaired vascular regulation [71,72]. Importantly, vascular dysfunction may persist despite improved glycemic control, highlighting the contribution of continuous cellular stress to diabetic vascular complications [73].

Hypertension similarly induces profound functional and structural changes in the vasculature, leading to hypercontractility, impaired relaxation, and vessel remodeling [74]. A major upstream contributor to these alterations is the activation of the renin–angiotensin–aldosterone system (RAAS), primarily via Ang II type 1 receptor (AT1R). Within the vasculature, Ang II-AT1R signaling, via enhanced Ca^2+^ mobilization, promotes vasoconstriction and oxidative stress, and it activates inflammatory pathways that contribute to vascular dysfunction and remodeling [53]. Mechanistically, altered Ca^2+^ handling is a central feature of hypertensive vasculature, as cytosolic Ca^2+^ concentration directly determines the degree of contraction and vascular tone. In hypertension, Ca^2+^ levels may be elevated due to increased influx through L-type Ca^2+^ channels, transient receptor potential (TRP) channels, store-operated channels (SOCs), and receptor-operated non-selective cation channels (ROCs), as well as dysregulated Ca^2+^ release from the SR through the IP3 receptor and ryanodine receptors (RyRs) [74,75]. These alterations increase VSMC contractility and vascular tone. Additionally, dysregulated STIM1/Orai-1-mediated store-operated Ca^2+^ entry has also been reported in hypertensive vasculature, contributing to hypercontractility [76,77]. In parallel, increased vasoconstriction can occur via enhanced Ca^2+^ sensitization independent of changes in cytosolic Ca^2+^. Of note, upregulation of Rho-kinase and PKC-mediated signaling suppresses MLCP activity and increases Ca^2+^ sensitivity, thereby promoting persistent vasoconstriction and peripheral resistance in hypertension [78,79].

Beyond the aforementioned pathways, evidence suggests that MAPK-associated pathways, including the p38 and extracellular signal-regulated kinase (ERK1/2), may also contribute to vascular dysfunction by enhancing vasoconstriction, hypertrophy, and proliferation, leading to remodeling and stiffness, which are further amplified by oxidative stress [80,81]. Additionally, increased ROS further amplifies these responses by reducing NO bioavailability, activating vasoconstrictor and pro-inflammatory signaling, disrupting VSMC Ca^2+^ homeostasis, and recruiting leukocytes [43,82]. Over time, these molecular alterations are translated into structural changes in the vascular wall. In resistance vessels, an increased wall-to-lumen ratio contributes to elevated peripheral resistance, whereas in larger conduit arteries, collagen accumulation, elastin fragmentation, and increased stiffness reduce vascular compliance [50,83]. In large vessels such as the aorta, abnormal Ca^2+^ handling is associated with vascular dysfunction and structural remodeling in hypertension [84]. Genetic predisposition may also influence hypertension-associated vascular dysfunction as variants affecting renal sodium handling (such as UMOD), natriuretic signaling (NPR3), vascular Ca^2+^ homeostasis (ATP2B1), inflammation (SH2B3), and endothelial signaling (NOS3) can mediate vascular dysregulation [85,86,87,88,89]. Although the impact of each common variant is small, their combined effect can increase susceptibility to hypertension.

**Figure 3:**
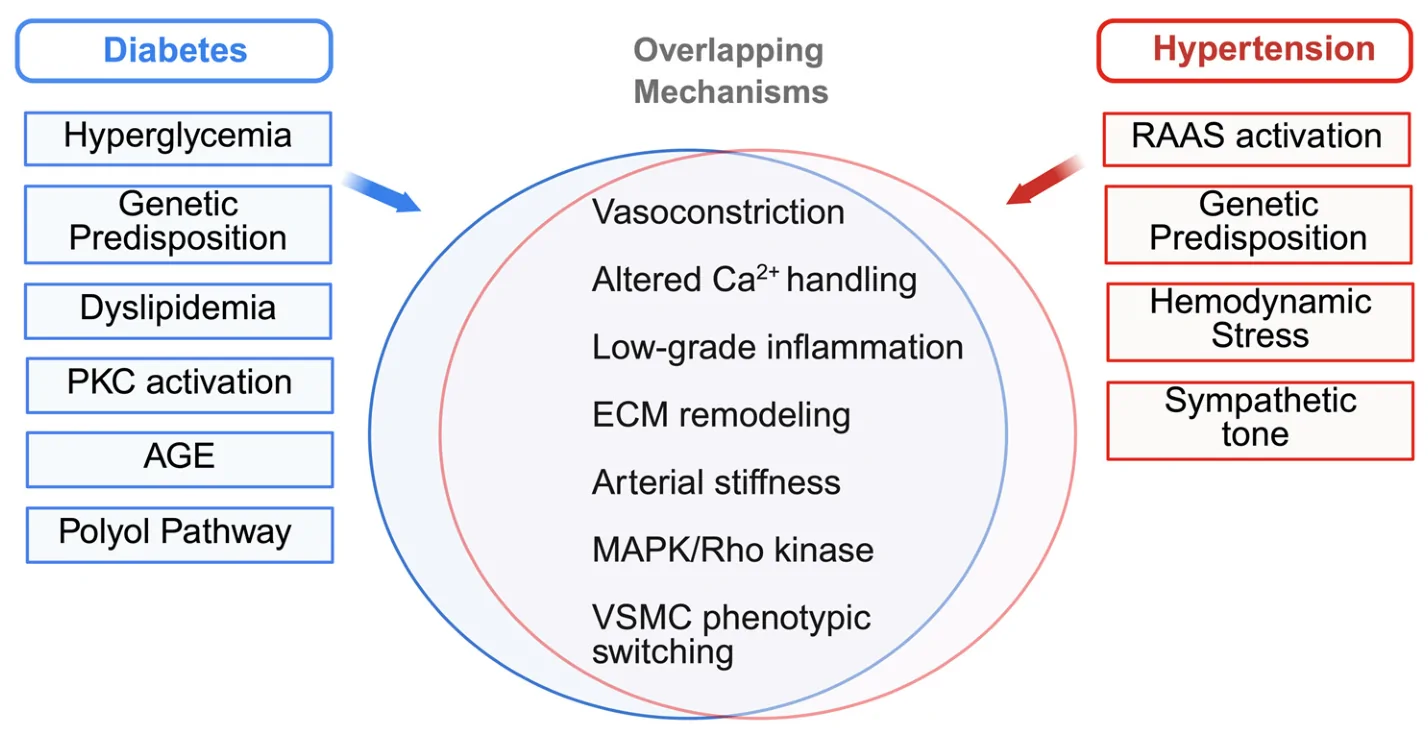
Converging mechanisms of diabetes and hypertension associated with vascular dysfunction. Diabetic metabolic disturbances are highlighted in the blue square, while hypertensive ones are in the red square. Circles in the middle show the overlapping mechanisms presented in both pathologies, such as vasoconstriction, abnormal Ca^2+^ handling, low-grade inflammation, and others. AGE: advanced glycation end-product; PKC: protein kinase C; ECM: extracellular matrix; MAPK: mitogen-activated protein kinase; Rho-kinase: rho-associated coiled-coil kinase; VSMC: vascular smooth muscle cell; RAAS: renin–angiotensin–aldosterone system. (Created in BioRender. https://BioRender.com/gce6vq3).

Taken together, although diabetes and hypertension arise from different metabolic and hemodynamic disturbances, many overlapping mechanisms are evident, converging on vascular damage, including oxidative stress, Ca^2+^ mishandling, increased RhoA/Rho-kinase-mediated Ca^2+^ sensitization, MAPK signaling, and reduced NO bioavailability. These mechanisms promote hypercontractility, impaired relaxation, and facilitate the transition of VSMCs from a differentiated contractile state to a synthetic and proliferative phenotype. Furthermore, it increases arterial stiffness and vascular remodeling by disrupting elastin–collagen balance [90]. All contribute to renal, retinal, cerebral, and other end-organ complications [91,92,93], thereby making vascular dysfunction a hallmark of both diseases. These overlapping mechanisms are summarized in Figure 3. Importantly, they also represent cellular stress that might trigger adaptive response mechanisms, including HSP70, which may influence vascular homeostasis and disease progression.

## 4. HSP70 in Diabetes: A Double-Edged Sword in the Vasculature

The widespread vascular dysfunction observed in diabetes is closely linked to increased cardiovascular risk, as chronic hyperglycemia disrupts multiple signaling pathways driving vascular complications [94]. Several studies now suggest that HSP70 is not merely a bystander but a potential mechanistic player in this setting [14,16,17,95]. Importantly, the available information derives from both experimental animal models and human observational studies, which provide complementary but distinct levels of evidence. Hypercontractility, a hallmark of diabetes, impairs vascular function and disrupts Ca^2+^ homeostasis [16,17]. Interestingly, accumulating evidence links HSP70 to Ca^2+^ handling under physiological conditions, as experimental studies of vascular function have shown that blocking HSP70 negatively impacts Ca^2+^ mobilization [31]. Notably, HSP70 can bind Ca^2+^ ions within its N-terminal ATPase domain at two specific sites [96]. In diabetes, combined data from diabetic patients and rodent models of different types of diabetes suggest that HSP70’s function depends on both location and relative abundance [16,17,97]. Reduced levels of iHSP70, associated with Ca^2+^ mobilization, have been reported in a T1D-induced animal model [16], whereas increased levels of eHSP70 correlate with longer diabetes duration in patients with T2D [97]. Furthermore, in the T2D model db/db, iHSP70 levels remain unchanged, whereas eHSP70 is elevated compared with non-diabetic controls [17].

There is a common belief that, in diabetes, increased iHSP70 expression in tissues is beneficial to cells, whereas elevated eHSP70 levels are detrimental [98]. However, this protective-versus-harmful distinction should be considered a context-dependent working model rather than a universal property of iHSP70 and eHSP70. While the debate seems to center on whether to promote iHSP70 expression or block eHSP70, or whether we are referring to a specific isoform, the focus has shifted to balancing these two to prevent low-grade inflammation. The Chaperone Balance Hypothesis posits that an imbalance between eHSP70 and iHSP70, quantified by the H-index, may serve as an inflammatory biomarker in diabetes [36]. The vascular consequences of this imbalance are likely shaped by the type of diabetes, sex, and disease duration. However, the relative contributions of these factors remain incompletely defined.

In a study using the streptozotocin-induced model of T1D, chronic hyperglycemia was associated with an increased eHSP70-to-iHSP70 ratio and H-index, altered Ca^2+^-dependent vascular responses, and activation of the innate immune system via the TLR4-MD2 complex located in blood vessels [16]. Notably, an interplay between the innate immune receptor TLR4/MD2 complex and HSP70 is reported in the human cardiovascular system, in which a proximity ligation assay for HSP70 and TLR4 shows that cells stimulated with ATP have higher formation of fluorescent spots and that MD2 is required for the complexation of these proteins [19]. Although these findings do not establish that eHSP70 is intrinsically harmful, they strongly support an association between an altered HSP70 profile and vascular dysfunction, especially given the consensus that TLR4 activation in the vasculature contributes to low-grade inflammation [90,99]. Thus, the effects of eHSP70 may also depend on the receptor’s type and the surrounding cellular environment. In T2D, in which insulin resistance, obesity, dyslipidemia, and chronic inflammation coexist, the same HSP70 pathways may be further modified by adipose-derived inflammatory mediators and endothelial metabolic dysfunction [36].

Together, the findings from the existing body of literature support the hypothesis that HSP70 should not be viewed as inherently protective or inherently harmful. Instead, its biological meaning in diabetes depends on whether one is measuring intracellular or extracellular pools, which tissue is being studied, and whether the system is in an adaptive or maladaptive phase of metabolic compensation. Importantly, in a db/db mouse model of T2D, pharmacological inhibition of HSP70 with VER155008 is beneficial by preventing abnormal vascular contractility and Ca^2+^ handling [17]. Given HSP70’s paradoxical role, one wonders how to best approach it. Therapeutically, selective inhibitors (e.g., VER 155008, JG98) show promise in reducing the inflammatory profile without compromising cytoprotection. However, neither VER155008 nor JG98 selectively target eHSP70 and may affect HSP70/HSC70-dependent pathways [100,101]. Thus, the lack of interventions that selectively distinguish eHSP70 from iHSP70 remains an important limitation when evaluating HSP70 as a therapeutic target.

An emerging concept from studies in both human diabetes and rodent models is that the relative balance between iHSP70 and eHSP70 may provide information regarding the biological state of the vasculature. However, it is essential to establish a baseline for comparing circulating and intracellular pools of this protein to identify the most biologically relevant therapeutic target. In the vascular context of T1D, studies report reduced iHSP70 alongside increased eHSP70 [16], whereas in T2D, other studies describe preserved iHSP70 with elevated eHSP70 [17]. Thus, in diabetes, HSP70 localization and balance may be differentially regulated according to disease type, progression, and underlying mechanisms.

**Figure 4:**
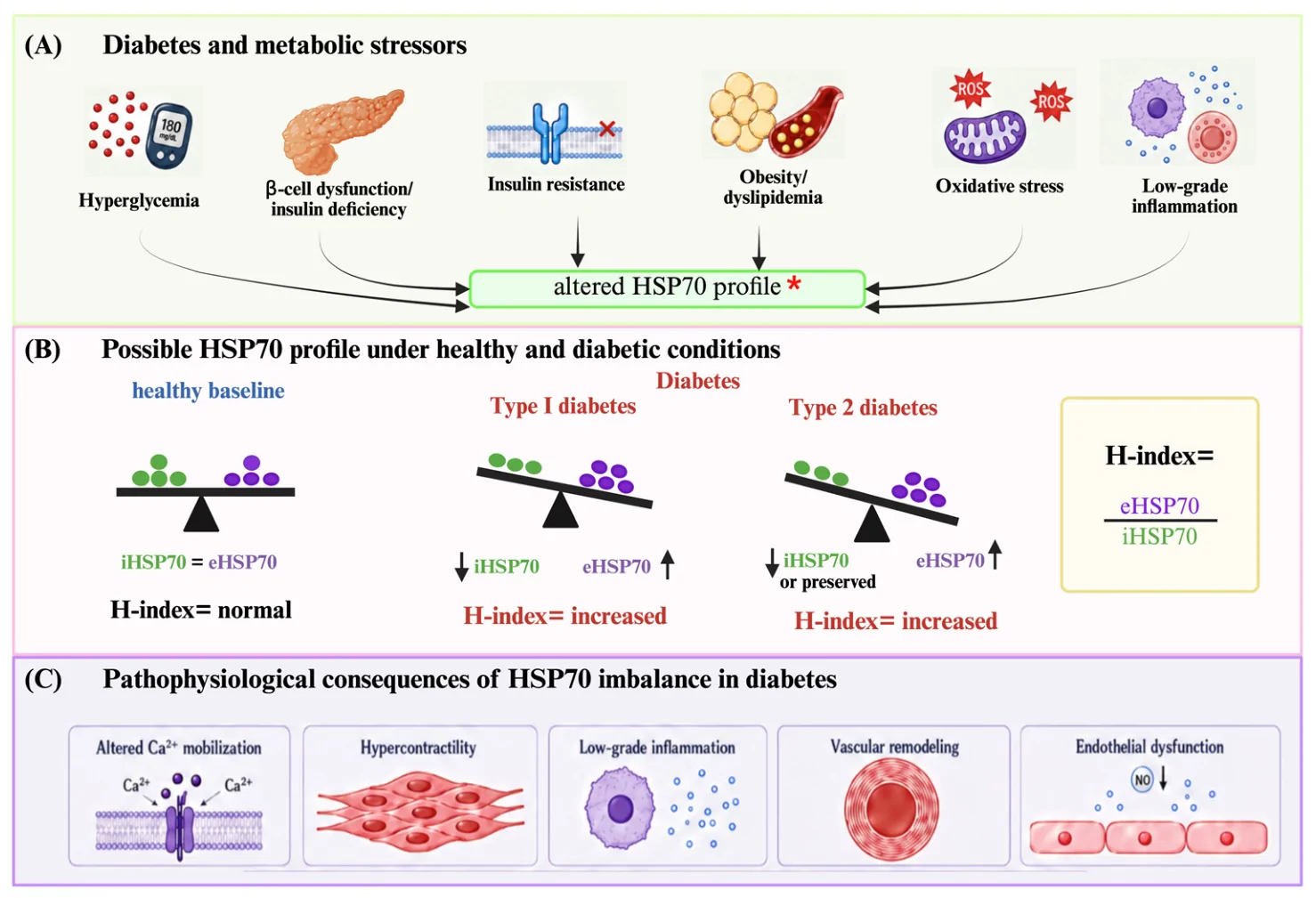
Metabolic stressors affecting the vasculature during diabetes, prospective HSP70 overall profile, and the consequences of HSP70 imbalance impacting the vasculature during diabetes. In panel A, diabetes-related metabolic stressors, including hyperglycemia, insulin deficiency or resistance, obesity/dyslipidemia, oxidative stress, and low-grade inflammation, lead to changes in HSP70. In panel B, presumed HSP70 levels and the H-index (eHSP70:iHSP70 ratio) under healthy and diabetic conditions are shown, based on data from the literature. HSP70 balance is context-dependent and may vary with diabetes type and sex. In panel C, a summary of effects triggered by an imbalanced HSP70 profile, such as dysfunctional Ca^2+^ mobilization, hypercontractility, inflammation, and vascular remodeling. *: context-dependent emerging evidence (Created in BioRender. Nunes, K. (2026) https://BioRender.com/z5mh28m).

Sex-related differences further complicate this picture. In young diabetic female mice (16 weeks), both iHSP70 and eHSP70 were reduced relative to non-diabetic controls, resulting in an unchanged eHSP70-to-iHSP70 ratio and H-index [102]. In humans, a similar sex-dependent pattern has been observed, with an increased eHSP70-to-iHSP70 ratio reported only in postmenopausal diabetic women, but not in premenopausal controls [20], corroborating the results observed in female diabetic mice. Moreover, elevated eHSP70 has been associated with longer diabetes duration [97] and aging [34], supporting the idea that eHSP70 reflects disease burden and progression. Figure 4 provides an overview of the pathophysiological stressors affecting HSP70 in the context of vascular dysfunction in diabetes, along with its profile reported in the literature. Table 2 summarizes reported HSP70 levels in humans and diabetic animal models, with sex considered a biological variable. Interestingly, recent studies have found that natural compounds, such as curcumin and berberine, may modulate HSP70 and reduce circulating eHSP70 levels in diabetes [103]. Curcumin also increased protective iHSP70 in the aorta of T1D diabetic rats. This coincided with improved elastin–collagen remodeling, supporting HSP70 profile balance as a potential therapeutic target in vascular dysfunction associated with diabetes [104]. However, the specific mechanisms linking these HSP70 changes to the vascular effects of these compounds remain unclear; therefore, the observed protective effects cannot be attributed entirely to HSP70 modulation.

## 5. The Dual Role of HSP70 in Hypertension

Resembling diabetes, vascular dysfunction is also a feature of hypertension. A central contributor to this process is the overactivation of RAAS. Ang II is a key molecule in this landscape, contributing not only to vasoconstriction but also to remodeling and vascular hypertrophy. These effects involve activation of oxidative and inflammatory pathways, including nuclear factor-κB (NF-κB), and the increased production of pro-inflammatory cytokines. Together, these underlying mechanisms disrupt homeostasis and activate stress-response pathways in the vessels [107]. In this context, HSP70’s relevance is highlighted, given that hypertension induces vascular stress, triggering a cellular response in which HSP70 may play a key role.

It is well known that HSP70 is a highly conserved protein and upregulated in response to various forms of cellular injury, including oxidative stress, hypoxia, environmental factors, and pathological conditions. Evidence shows that HSP70 has also been implicated in hypertension [18,23]. Earlier experimental studies in animals and humans with hypertension have shown that HSP70 mRNA expression increases during acute stress, but this response may decline with chronic stress exposure [108,109,110]. Udelsman et al. showed that restraint stress increases iHSP70 expression in the murine aorta through α1-adrenergic-dependent stimulation, suggesting that vascular iHSP70 is regulated not only by the classical heat shock pathway but also by receptor-mediated responses in mice, thereby providing a direct link between adrenergic stimulation and vascular HSP70 expression [111]. Later, a study extended this information within the hypertensive panorama by demonstrating that both acute restraint and jet air-induced stress elevated systolic blood pressure and aortic iHSP70 mRNA expression in rats exposed to acute stress [112]. Similarly, further reports corroborated the results for acute restraint, a response secondary to the pressure rise, and showed that increased blood pressure and iHSP70 expression were prevented when the pressure rise was blocked with sodium nitroprusside in rat aorta [113]. These animal experiments support a relationship between acute elevation of blood pressure and induction of vascular iHSP70, suggesting that HSP70 is responsive to hemodynamic stress, although its functional consequences may depend on the cellular and physiological context.

Human occupational studies further support an association between anti-HSP70 antibodies and hypertension, showing higher levels of HSP70 autoantibodies in workers exposed to harsh conditions, particularly among individuals with more severe increases in blood pressure values [114]. This observational study supports an association among anti-HSP70 autoantibodies, occupational stress, and elevated blood pressure, but does not establish a role of HSP70 in hypertension. Overall, these findings support further investigation of HSP70 in hypertension, particularly in understanding whether its response remains protective or becomes part of a dysfunctional vascular system.

Recent investigations have explored the role of iHSP70 in Ang II-mediated vascular damage. In murine VSMCs, increased HSP70 levels attenuated Ang II-induced hypertrophy by inhibiting ERK1/2 signaling via MAPK phosphatase-1 (MKP-1) [22]. This mechanistic evidence at the cellular level suggests a protective role for iHSP70 against Ang II-induced VSMC hypertrophy and remodeling. By showing that iHSP70 can suppress ERK1/2 in a scenario driven by Ang II upregulation, HSP70 may act upstream of this pathway, protecting against pathological vascular damage in hypertension. Similarly, activation of the heat shock response in vivo, accompanied by increased aortic HSP70 and HSP27 expression, attenuated Ang II-induced hypertension and vascular inflammation. This effect was associated with NF-κB suppression and reduced levels of pro-inflammatory cytokines, such as IL-6 [115]. Because the intervention induced multiple heat shock proteins, it does not establish that iHSP70 alone was responsible for the reduction in blood pressure or inflammatory response.

The cytoprotective role of iHSP70 extends beyond structural remodeling and may also involve regulation of oxidative stress and cytoskeletal organization. Ang II stimulates NADPH oxidase activity, including Nox4/p22phox, thereby increasing ROS production and promoting cytoskeletal reorganization. In VSMCs derived from spontaneously hypertensive rats (SHRs), AT1 receptor blockade with losartan increases iHSP70 expression while reducing Nox4 and p22phox, NADPH oxidase activity, and actin stress fiber formation [116]. Notably, silencing HSP70 diminished these effects, indicating that iHSP70 contributes to losartan’s protective effects by limiting oxidative and structural changes in hypertensive VSMCs [116]. Therefore, this cell-based study provides mechanistic evidence that HSP70 contributes to the cellular response to AT1-receptor blockade in VSMCs from a chronic genetic hypertension model. This represents a cell-based mechanistic model of hypertension-associated vascular dysfunction; however, because it was derived from isolated cells, it has translational limitations that warrant in vivo investigation. Similar findings in renal proximal tubules from SHR have been reported, showing that AT1R blockade with losartan promotes iHSP70 translocation to the plasma membrane and its colocalization with caveolin-1, thereby reducing oxidative stress, epithelial–mesenchymal transition, and fibrosis by inhibiting matrix metalloproteinases-2 and -9 (MMP-2 and MMP-9) [117,118]. Although these studies were conducted in renal tissue rather than in the vasculature, they provide additional evidence for the cytoprotective role of iHSP70 in hypertension-associated tissue damage.

HSP70 may also contribute to endothelial adaptation to hemodynamic stress. ECs are continuously exposed to mechanical forces, particularly shear stress, which regulate critical cell signaling for vascular homeostasis. Mechanistically, studies using primary human endothelial cells show that iHSP70 interacts with junctional proteins such as VE-cadherin and PECAM-1, thereby regulating eNOS levels, endothelial permeability, and barrier function [119]. However, this response may be disrupted under pathological conditions. In ECs derived from patients with chronic thromboembolic pulmonary hypertension, basal iHSP70 expression was reduced, and shear stress failed to increase HSP70. This impaired response was associated with disrupted EC migration, which was further exacerbated by pharmacological inhibition of iHSP70 [120]. Collectively, these results indicate that HSP70 supports vascular homeostasis by helping ECs adapt to shear stress and that impaired iHSP70 induction may contribute to endothelial dysfunction in hypertensive conditions [120], a first step in the progression of vascular dysfunction. However, direct evidence linking this mechanism to systemic hypertension-associated endothelial dysfunction remains limited.

Considering that iHSP70 appears to tend to be predominantly cytoprotective to date, its extracellular form in circulation may exert more complex biological effects. This is because once released systemically, eHSP70 may no longer function as a chaperone but instead act as a damage-associated molecular pattern (DAMP), interacting with innate immune receptors such as TLRs. This interaction can promote pro-inflammatory signaling and ECM remodeling, both of which are major contributors to hypertension-associated vascular dysfunction. Pockley et al. reported no significant difference in circulating HSP70 levels between individuals with established hypertension and the normotensive group [121]. However, the anti-HSP70 antibody levels were elevated in hypertensive patients, providing a possible association between the disease and altered HSP70-related immune responses [121]. Conversely, a later report showed increased circulating eHSP70 in the serum of patients with essential hypertension, which positively correlated with IL-6, TNF-α, and C-reactive protein, suggesting its involvement in systemic inflammation [23]. These findings suggest an association between eHSP70 and the inflammatory milieu of hypertension, but do not establish that eHSP70 causes systemic inflammation or hypertension.

Further studies support the potential role of eHSP70 in vascular remodeling. In human aortic VSMCs, eHSP70 has been shown to promote transforming growth factor beta 1 (TGF-B1)-dependent fibronectin and collagen synthesis via TLR4 [122]. These findings mechanistically support the notion that eHSP70 may promote profibrotic signaling in the vasculature, thereby contributing to ECM remodeling, a major determinant of vascular stiffening in hypertension [122]. However, evidence from non-vascular cells suggests that eHSP70 may not always be pro-inflammatory and may act differently depending on downstream signaling and cellular context [123]. For instance, in human bronchial epithelial cells, eHSP70 exerted anti-inflammatory effects via TLR4 signaling and attenuated inflammatory interleukins such as IL-6 and IL-8, demonstrating that HSP70 effects may be receptor- and tissue-specific [123]. Although this evidence comes from airway epithelial cells rather than vascular cells, it further highlights the variable biological effects of eHSP70.

HSP70 becomes even more significant when considering immune-mediated mechanisms of hypertension beyond the vasculature. HSP70 appears to facilitate antigen presentation and modulate T-cell responses. In the salt-sensitive hypertension model, HSP70 can act as an autoantigen in the kidney, where immune reactivity against HSP70 promotes inflammation and impairs sodium handling, thereby elevating blood pressure [124]. Although the mechanism suggested in this study involves renal immune responses rather than the direct vascular effects of HSP70, the kidney is highly vascularized, which does not rule out the possibility that HSP70 activation is multifunctional.

**Figure 5:**
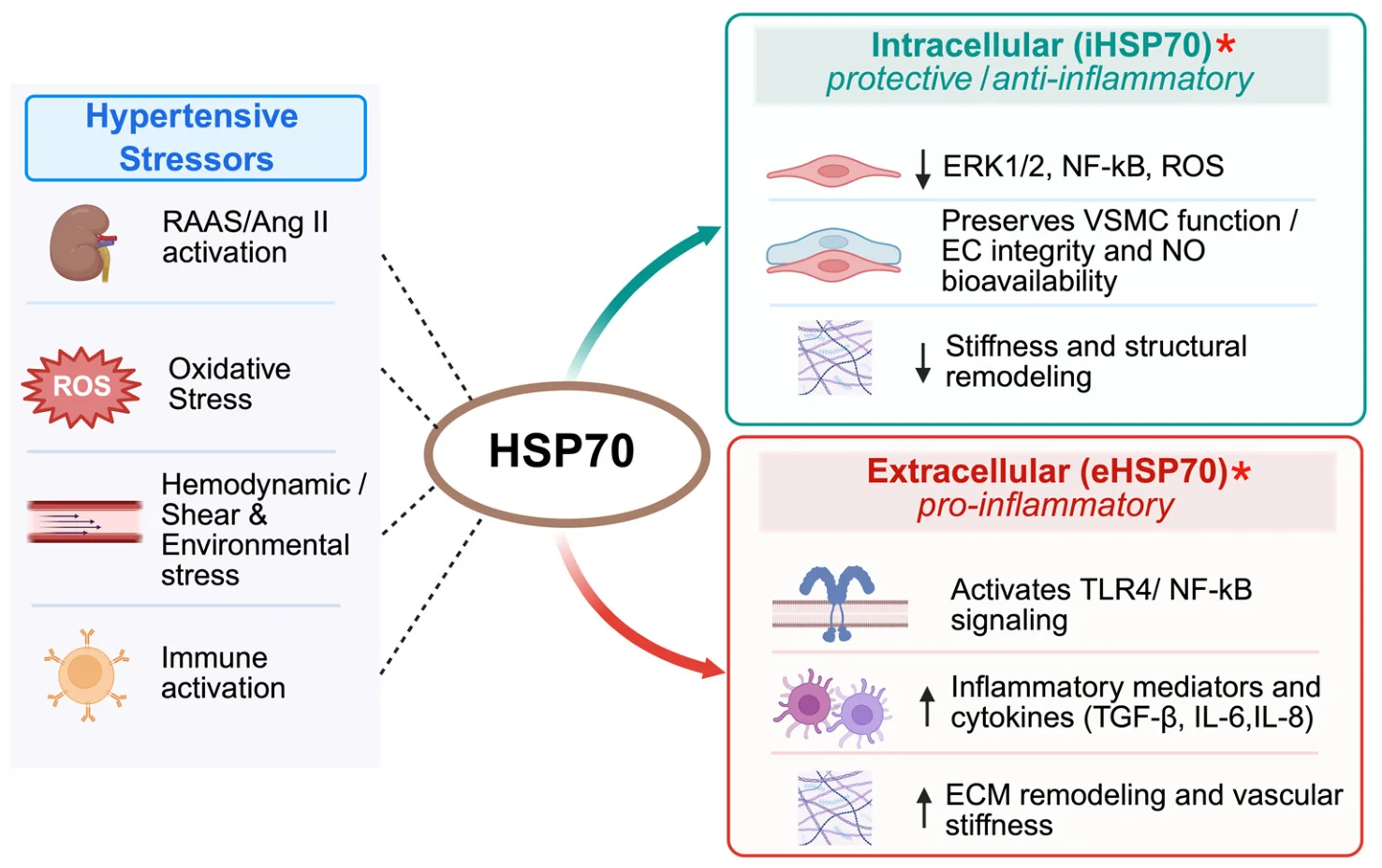
An overview of different types of distress linked to the HSP70 expression and modulation of this protein in different milieus. The green square summarizes the protective effects of HSP70 in VSMCs, while the red square highlights the pro-inflammatory effects of circulating HSP70. *: context-dependent emerging evidence. RAAS: renin–angiotensin–aldosterone system; Ang II: angiotensin II; ERK1/2: extracellular signal-regulated kinases 1 and 2; NF-kB: nuclear factor kappa B; VSMC: vascular smooth muscle cell; EC: endothelial cell; ROS: reactive oxygen species; NO: nitric oxide; TLR4: toll-like receptor 4; TGF-β: transforming growth factor beta; IL-6: interleukin-6; IL-8: interleukin-8; ECM: extracellular matrix (Created in BioRender. https://BioRender.com/a04wzvo).

Based on the literature, HSP70 is a plausible mediator of vascular, oxidative, and immune disturbances in hypertension. iHSP70 is generally associated with a cytoprotective profile that stabilizes vascular signaling pathways involved in oxidative stress and endothelial mechanisms, but there is no direct evidence that HSP70 mediates hypercontractility during hypertension, as it is described in diabetes. Conversely, eHSP70 might induce pro-inflammatory responses or remodeling in both pathologies, although these effects are highly context-dependent and can be modulated by different signaling pathways (Figure 5). Notably, although HSP70 has been extensively studied in other metabolic diseases, its role in vascular dysfunction-associated hypertension remains underexplored, with much of the recent literature heavily focused on the immune system. Many questions remain, such as whether blockade of iHSP70 could help reduce hypercontractility or if HSP70 would have sex-specific effects in hypertension. Given that the development and progression of CVD differ between males and females [125] and that tissue HSP70 responses are sex-dependent [33], addressing these gaps may help determine the therapeutic potential of targeting HSP70 to mitigate vascular damage. Nevertheless, we identified several relevant studies linking HSP70 to hypertension and compiled their findings in Table 3, taking sex as a biological variable.

## 6. Future Perspectives and Clinical Trials

As science is an ever-evolving field, emerging technologies could provide the means to investigate the cellular, molecular, and functional contexts in which HSP70 contributes to vascular health and disease. Integration of multi-omics (such as genomics, transcriptomics, proteomics, metabolomics, epigenomics, fluxomics, and lipidomics) could help determine whether alterations in HSP70 are accompanied by changes in inflammation, oxidative stress, and metabolic pathways, thereby distinguishing adaptive or maladaptive changes associated with the chaperone. Although complex and promising, this multi-omics technology network is starting to mold the field of CVDs [127,128]. Single-cell and spatial omics approaches may be valuable for investigating HSP70 cell-specific responses within the vascular wall [129,130]. Such approaches could clarify and further differentiate the roles of systemic versus cellular HSP70 and evaluate distinct molecular signatures that characterize different vascular pathologies and biological sex.

An important translational challenge is establishing whether HSP70 is merely a marker of vascular stress or a therapeutically actionable determinant of vascular dysfunction. Pharmacological modulation offers a potentially useful strategy, although the biological consequences depend on the mechanism, cellular compartment, and degree of HSP70 modulation. However, the molecular processes underlying these effects remain incompletely elucidated, and further research is needed.

Geranylgeranylacetone (GGA), for instance, is an antiulcer medication and an inducer of HSP70 at specific concentrations, with demonstrated cytoprotective and cardiovascular effects in experimental animal models, across different tissues, including the heart [131], liver [132], kidneys [133], and gastric mucosa [134]. GGA has also been investigated in models of metabolic dysfunction, including mice with diet-induced insulin resistance, where HSP70 induction was associated with improved insulin sensitivity [135]. However, its actions are not exclusively mediated by HSP70, and there is insufficient evidence that GGA does not affect other HSP family members [136]. Importantly, pharmacological regulators of HSP70 have progressed beyond preclinical studies, with some agents now being evaluated in human clinical trials, although no HSP70-directed drug has yet to demonstrate clinical efficacy in CVDs. Nevertheless, the human intervention studies summarized in Table 4 provide an important framework for preliminary evidence of the feasibility and tolerability of HSP70 modulation via pharmacological agents.

Although these studies were conducted in heterogeneous clinical settings and were not specifically designed to evaluate CVD outcomes, their findings demonstrate that HSP70-related responses could be pharmacologically modified in humans. The direction and magnitude of these effects varied considerably among interventions: tanespimycin induced a marked dose-dependent increase in HSP70 [137,138], whereas piroxicam reduced circulating HSP70 [139,140], and saffron supplementation decreased anti-HSP70 antibody levels [141]. In contrast, arimoclomol did not significantly alter muscle HSP70 compared with placebo [142,143], and enzyme-treated asparagus extract produced a non-significant increase in HSP70 mRNA expression [144]. These findings are promising and provide valuable evidence of pharmacological modulation of the HSP70 system in humans, while also highlighting its context-dependent nature and the need for adequately powered trials specifically targeting cardiometabolic and vascular outcomes.

Ultimately, future studies should combine mechanistic experiments, longitudinal clinical cohorts, multi-omics profiling, and pharmacological interventions to determine whether HSP70 can serve as a modifiable driver of vascular dysfunction in CVDs. Meticulous attention should be given to sex-specific differences in the molecular and vascular aspects of CVD pathophysiology, as these factors may determine whether HSP70 inhibition or activation, or even the promotion of the eHSP70/iHSP70 balance, can prove beneficial. Such an integrated approach could transform HSP70 from a broadly measured stress marker into a biologically informed key biomarker and a promising, viable therapeutic approach within precision-targeted cardiovascular medicine. Clarifying these mechanisms is particularly important given the substantial and growing global burden of hypertension and diabetes, two major and frequently coexisting cardiometabolic disorders that contribute substantially to vascular dysfunction and cardiovascular morbidity and mortality. A better understanding of whether HSP70 represents a causal mediator, compensatory response, or biomarker of vascular injury under these conditions, either independently or through synergistic interactions, may therefore have broad clinical relevance and could facilitate the identification of novel therapeutic strategies for populations at high cardiovascular risk.

**Table 4:** Human intervention studies reporting HSP70 levels. * Nutraceutical/food-derived intervention. ** Mean (Standard Deviation). CNS: central nervous system; mRNA: messenger ribonucleic acid; ISRCTN: International Standard Randomized Controlled Trial Number; NCT: ClinicalTrials.gov Identifier; IRCT: Iranian Registry of Clinical Trials; ETAS: enzyme-treated asparagus extract.

Trial Registration	Study Approach	Intervention (n)	Study design	HSP70 Assessment
NCT00769860 ISRCTN80057573 [143]	Arimoclomol in Sporadic Inclusion Body Myositis	Arimoclomol (n = 15) Placebo (n = 8)	Interventional	HSP70 measurement in muscle biopsies at the baseline and after 4 months of treatment
	Main HSP70 outcome	• Arimoclomol: −110.72(757.40) ** • Placebo: −34.70(336.35) ** • No significant difference between groups		
IRCT2017012132081N2 [141]	A triple-blind, randomized placebo-controlled trial: an evaluation of the effect of saffron supplementation on the antibody titer to HSP70, hsCRP, and spirometry test in patients with mild and moderate persistent allergic asthma	Saffron (n = 38) Placebo (n = 38)	Interventional	Anti-HSP70 antibody measurement in asthma patients under supplementation with saffron at baseline and 8 weeks post-intervention
	Main HSP70 outcome	• Saffron, in comparison to placebo, decreased the serum levels of anti-HSP70 significantly • Saffron: −0.06(0.24) ** • Placebo: 0.46(0.27) ** p value < 0.001		
No trial registration identified * [144]	Effects of Enzyme-treated Asparagus Extract on heat shock protein 70, stress indices, and sleep in healthy adult men	ETAS (n = 10) Placebo (n = 10)	Interventional	Assessment of enzyme-treated asparagus extract (ETAS) HSP70 mRNA expression in blood and CNS at the baseline and after 7 days of treatment
	Main HSP70 outcome	• In both groups, the expression of HSP70 mRNA was higher than the baseline, and the expression level was greater in the ETAS group, but the difference was not statistically significant • *p* value = 0.98		
NCT00514371 [137,138]	Tanespimycin with bortezomib: activity in relapsed/refractory patients with multiple myeloma	Tanespimycin + bortezomib (n = 10)	Interventional	HSP70 measurement in patients with multiple myeloma treated with tanespimicyn + bortezomib in multiple doses
	Main HSP70 outcome	• HSP70 levels increased according to the dose: the major increase occurred with the 340 mg dose (26.5-fold)		
ISRCTN58517443 [139,140]	Immunomodulatory effect of NSAID in geriatric patients with acute infection: effects of piroxicam on chemokine/cytokine secretion patterns and levels of heat shock proteins: A double-blind randomized controlled trial	Piroxicam (n = 15) Placebo (n = 15)	Interventional	Cytokines and HSP70 measurement in geriatric patients with acute infection receiving piroxicam, at baseline and after 10 days
	Main HSP70 outcome	• The serum levels of HSP70 decreased significantly in the piroxicam group • *p* value < 0.05		

## 7. Conclusions

Although substantially more data are available on diabetes than on hypertension, the current literature supports a role for HSP70 in vascular function. The increased levels of eHSP70 are relatively well established in both diabetes and hypertension, with growing evidence linking it to low-grade inflammation and immune activation. In contrast, iHSP70 is generally associated with cellular protection through the maintenance of proteostasis and the attenuation of oxidative and pro-inflammatory stress. However, much of the evidence supporting these effects derives from experimental models, and the extent to which these mechanisms operate in human vascular disease remains uncertain.

Several findings provide a basis for further investigation of iHSP70 in vascular dysfunction. iHSP70 has been implicated in the regulation of Ca^2+^ handling and contractile function in diabetes, whereas comparable evidence in hypertension remains limited. Although the mechanisms by which HSP70 regulates Ca^2+^ handling during vascular contraction have been characterized under physiological conditions, it remains unclear whether the same mechanisms are affected by HSP70 in diabetes and hypertension. For example, HSP70 blockade in the aorta affects IP3R-mediated Ca^2+^ release and voltage-independent Ca^2+^ channels, facilitating Ca^2+^ influx, without apparent crosstalk with Rho-kinase in the non-pathological state [31]. Whether this relationship changes when Rho-kinase signaling is upregulated, as occurs in both diabetes and hypertension, remains unknown.

Sex is another potentially important determinant of HSP70-mediated vascular responses. The greater extent of differences in the CVD profile and risk factors in women has been increasingly recognized, and experimental evidence indicates that female vasculature is more sensitive to HSP70 [33]. Interestingly, the female heart has twice as much HSP70 as the male counterpart, suggesting a possible cardioprotective effect that might be lost following ovariectomy [145]. Additionally, sex-related differences in Ca^2+^ signaling have been noted in hypertension, with females exhibiting reduced Ca^2+^ influx [146]. These observations suggest that HSP70 might contribute differently to cardiovascular responses across sexes. Still, whether HSP70 influences vascular responses to diabetic or hypertensive stress in a sex-dependent manner remains to be elucidated. Moreover, the translational relevance of these experimental findings in animal models requires further investigation.

Notably, correlating basal HSP70 levels with distinct pathological scenarios could help clarify whether HSP70 serves as a biomarker for assessing vascular health, despite uncertainties regarding its role as a therapeutic target. To date, selectively reducing detrimental extracellular HSP70 is strongly suggested to mitigate low-grade inflammation, but the concept of how modulating tissue-level HSP70 would be beneficial remains underdeveloped. At present, however, this challenge remains largely preclinical, and strategies that selectively modulate both pools of HSP70 have not been sufficiently validated in patients with diabetes or hypertension. Also, whether these associations differ between the two pathologies requires further investigation. Addressing these questions will be necessary before HSP70 can be considered a validated therapeutic target or biomarker in CVDs.

## 8. Literature Search

This narrative review was conducted using a structured literature search of PubMed/MEDLINE, Scopus, and Google Scholar. The literature search covered publications from inception to August 2026. Searches were performed using a combination of terms related to HSP70 and its cellular compartments, as well as terms related to cardiovascular diseases, diabetes, hypertension, endothelial dysfunction, vascular smooth muscle cells, and pharmacological modulation of HSP70. Relevant primary research articles, clinical studies, and review articles were selected based on their relevance to the scope and mechanistic focus of the review. As this was a narrative review rather than a systematic review or meta-analysis, the literature search aimed to identify and synthesize key evidence relevant to the central themes, rather than to provide an exhaustive assessment of all available or ongoing studies.

## Figures and Tables

**Table 1:** HSP70 nomenclature and terminology used throughout this review. Summary of the HSP70-related isoforms and terms referenced in this review, including their corresponding nomenclature, expression, and localization.

Terms	Meaning
HSP70	A collective term used for the HSP70/HSPA family
HSPA1A/HSPA1B	Stress-inducible HSP70 family member
HSC70/HSPA8	Constitutive form of HSP70
iHSP70 (Intracellular HSP70)	Refers to the location of the chaperone (inside the cell and tissue)
eHSP70 (Extracellular HSP70)	Refers to the location of the chaperone (circulation and fluids)

**Table 2:** Summary of HSP70 profiles in diabetes across human and animal models. The table highlights HSP70 levels in serum and aortic tissue across different sexes in both T1D and T2D disease models.

Reference	Diabetes Model (n)	Location	Sex	HSP70 Levels
Yabunaka et al. (1995) [105]	T2D Patients (Control n = 12; Diabetic n = 12)	Serum (peripheral blood mononuclear cells)	Both	iHSP70 is elevated in diabetic patients
	Main findings	• HSP70 levels were significantly higher in diabetic patients • iHSP70 and age were inversely (negatively) correlated		
	Assay	Western blot with laser densitometry		
	Main limitation	Small sample size (12 T2D patients and 12 controls), limiting generalizability		
Nakhjavani et al. (2010) [97]	T2D Patients (Control n = 36; Diabetic n = 73)	Serum	Both	Serum HSP70 levels are significantly elevated and correlated with oxidative stress markers
	Main findings	• HSP70 levels were higher in patients with long-standing diabetes (more than 5 years) • HSP70 and age were positively correlated		
	Assay	Quantitative sandwich ELISA (EKS-700B, Stressgen)		
	Main limitation	Cross-sectional design, precluding determination of the direction of causality		
Nakhjavani et al. (2011) [106]	T2D Patients (Control n = 18; Diabetic n = 18)	Serum	Both	Serum HSP70 levels are higher in T2D patients
	Main findings	• Serum HSP70 was higher in T2D than in controls and higher in long-standing diabetes • Women with long-standing diabetes had higher HSP70 than men • Metformin reduced HSP70 in men with newly diagnosed diabetes, but not in women		
	Assay	Quantitative sandwich ELISA (EKS-700B and EKS-715, Stressgen)		
	Main limitation	Use of the EKS-700B kit for serum HSP70 measurement, although it was not validated by the manufacturer for serum samples		
de Oliveira et al. (2022) [16]	STZ-induced rat (T1D) (Total n = 50; Diabetic n = 20)	Thoracic aorta and serum	Male	iHSP70 is decreased; eHSP70 linked to TLR4-MD2 activation is increased
	Main findings	• Reduced iHSP70 levels impact the vessel’s ability to handle Ca^2+^ from intra- and extra-cellular stores • Increased eHSP70 levels affect the functionality of arterial structures by potentially modulating the TLR4-MD2 complex		
	Assay	Western blot, ELISA, and wire myography		
	Main limitation	Ca^2+^ dynamics were assessed indirectly through vascular force development rather than by direct measurement of intracellular Ca^2+^		
Seibert et al. (2022) [20]	Prediabetic and T2D postmenopausal women (Control n = 18; Diabetic n = 18)	Plasma (peripheral blood mononuclear cells)	Female	The eHSP70-to-iHSP70 ratio was only increased in T2D postmenopausal women
	Main findings	• In postmenopausal women, the glycemic status does not affect eHSP70, but the eHSP70-to-iHSP70 ratio is increased only in a diabetic state, not in normoglycemic subjects		
	Assay	ELISA for eHSP70 and iHSP70; calculation of the eHSP70/iHSP70 ratio (H-index)		
	Main limitation	Small sample size and potential influence of medications and comorbidities		
Ochoa Mendoza et al. (2025) [17]	db/db T2D mouse (Control n = 5–7; Diabetic n = 5–7)	Thoracic aorta and serum	Male	iHSP70 did not change, whereas eHSP70 was elevated in serum
	Main findings	• HSP70 inhibition reduced the abnormal vascular contraction observed in T2D		
	Assay	Western blotting, immunofluorescence, wire myography, and biochemical Ca^2+^ assay		
	Main limitation	Use of a single pharmacological HSP70 inhibition method (VER155008), which targets multiple HSP70 isoforms		
Ochoa Mendoza et al. (2026) [103]	db/db T2D mouse (Control n = 9; Diabetic n = 5)	Thoracic aorta and serum	Male	Berberine treatment lowered iHSP70 and eHSP70, resulting in an unchanged ratio
	Main findings	• Long-term Berberine treatment prevents hypercontraction-related diabetes		
	Assay	Wire myography, Western blotting, and ELISA		
	Main limitation	Endothelium removal limited the evaluation of endothelial contributions to berberine-induced vascular effects		
Rastogi et al. (2025) [104]	STZ-induced rat (T1D) (Control n = 6; Diabetic n = 6)	Thoracic aorta and serum	Male	eHSP70/iHSP70 is disrupted in diabetic animals
	Main findings	• Long-term curcumin treatment restored the HSP70 profile and improved vascular function		
	Assay	Wire myography, Western blotting, ELISA, Picrosirius staining, and fluorescence microscopy		
	Main limitation	Indirect measurement of Ca^2+^ mobilization		

**Table 3:** Summary of HSP70 profiles in hypertension across human and animal models. The table highlights HSP70 levels in serum and various tissues across sexes and types of hypertension. * Sample size not disclosed.

Reference	Hypertension Model	Location	Sex	HSP70 Levels
Kuneš et al. (1992) [110]	Essential hypertension patients (Normotensive n = 11; Hypertensive n = 11)	Peripheral blood lymphocytes	Both	Heat stress induced greater HSP70 mRNA accumulation in hypertensive patients
	Main findings	• Hypertension was associated with an enhanced HSP70 response to heat stress		
	Assay	Ex vivo heat-stress assay and measurement of HSP70 mRNA expression in peripheral blood lymphocytes		
	Main limitation	Small sample size (11 hypertensive and 11 normotensive subjects)		
Xu et al. (1995) [113]	Essential hypertension in SHR	Thoracic aorta	Male	HSP70 mRNA and protein increased following acute elevation of BP
	Main findings	• Acute hypertension induced HSP70 expression in the aortic wall, suggesting a stress response to increased hemodynamic load		
	Assay	HSP70 mRNA and protein expression analysis following pharmacologically induced acute hypertension		
	Main limitation	The acute hypertension model limits extrapolation to chronic hypertension		
Pockley et al. (2002) [121]	Established hypertension patients (Normotensive n = 75; Hypertensive n = 111)	Serum	Male	HSP70 levels were similar between hypertensive and normotensive subjects
	Main findings	• Circulating HSP70 was not altered in established hypertension; therefore, HSP70 itself was not associated with hypertension in this cohort		
	Assay	Enzyme immunoassay for circulating HSP70 and anti-HSP70 antibodies; carotid ultrasonography		
	Main limitation	The study included only men and no sex-specific differences in circulating HSP70 were assessed.		
Pockley et al. (2003) [126]	Established hypertension patients (Hypertensive n = 218)	Serum	Both	Higher serum HSP70 is associated with less progression of carotid thickness and atherosclerosis
	Main findings	• Subjects with higher baseline HSP70 had significantly less progression of carotid atherosclerosis over 4 years, suggesting a potentially protective association		
	Assay	Enzyme immunoassay for serum HSP70 and carotid ultrasonography for intima-media thickness.		
	Main limitation	The observational design does not establish a causal protective role of HSP70 in atherosclerosis progression		
Srivastava et al. (2016) [23]	Newly diagnosed essential hypertension patients (Normotensive n = 132; Hypertensive = 132)	Serum	Both	HSP70 mRNA and circulating HSP70 protein were increased in hypertensive patients
	Main findings	• Elevated HSP70 was associated with inflammatory markers, including IL-6, TNF-α and CRP		
	Assay	qRT-PCR, Western blotting, and indirect ELISA		
	Main limitation	The cross-sectional design limits conclusions regarding causality between HSP70 expression, inflammation, and hypertension		
Pons et al. (2012) [124]	Induced salt-sensitive hypertension in rats (Normotensive n = 20; Hypertensive n = 20)	Kidney	Male	Renal HSP70 expression was increased in salt-sensitive hypertensive rats
	Main findings	• Increased renal HSP70 was associated with the development of salt-sensitive hypertension and renal inflammatory responses		
	Assay	Western blotting, immunohistochemistry, T-cell proliferation assay, and blood pressure measurement		
	Main limitation	The findings from this experimental model require validation in other models of salt-sensitive hypertension and in humans		
Gil Lorenzo et al. (2013) [116]	Essential hypertension in SHR *	VSMCs derived from mesenteric resistance arteries	Male	iHSP70 increased in the plasma membrane following losartan treatment
	Main findings	• Losartan treatment promoted elevated iHSP70 translocation to plasma membrane • HSP70 is required for the antioxidative and cytoskeleton modulation effects of AT1R inhibition • HSP70 acts as a negative regulator of Nox4/p22phox after losartan treatment in VSMCs from SHR		
	Assay	Western blotting, immunoprecipitation, confocal immunofluorescence microscopy, and Hsp72 knockdown		
	Main limitation	The use of isolated cultured VSMCs limits extrapolation of the findings to intact vascular function in vivo		

## Data Availability

No new data were created or analyzed in this study. Data sharing is not applicable to this article.

## Abbreviations

ADP	Adenosine diphosphate
AGEs	Advanced glycation end-products
Ang II	Angiotensin II
ATP	Adenosine triphosphate
AT1R	Angiotensin II type 1 receptor
CaM	Calmodulin
cGMP	Cyclic guanosine monophosphate
CVD	Cardiovascular disease
DAG	Diacylglycerol
db/db	Mouse model of type 2 diabetes
ECM	Extracellular matrix
EC	Endothelial cell
eHSP70	Extracellular heat shock protein 70
eNOS	Endothelial nitric oxide synthase
ER	Endoplasmic reticulum
ERK1/2	Extracellular signal-regulated kinases 1 and 2
GGA	Geranylgeranylacetone
GPCR	G-protein coupled receptors
GTP	Guanosine triphosphate
HSF	Heat shock factor
HSP	Heat shock protein
HSP70	Heat shock protein 70
iHSP70	Intracellular heat shock protein 70
IL-6	Interleukin 6
IP3	Inositol 1,4,5-triphosphate
IP3R	Inositol 1,4,5-triphosphate receptor
MHC	Major histocompatibility complex
MLC	Myosin light chain
MLCK	Myosin light chain kinase
MLCP	Myosin light chain phosphatase
MMP	Metalloproteinase
MCP-1	Monocyte Chemoattractant Protein-1
NADPH	Nicotinamide adenine dinucleotide phosphate
NBD	Nucleotide binding domain
NEF	Nucleotide exchange factor
NF-kB	Nuclear factor kappa B
NO	Nitric oxide
PIP2	Phosphatidylinositol 4,5-bisphosphate
PKA	Protein kinase A
PKC	Protein kinase C
PKG	Protein Kinase G
PLC	Phospholipase C
RAAS	Renin–angiotensin–aldosterone system
Rho-Kinase	Rho associated coiled-coil kinase
RhoA	Ras homolog family member A
ROS	Reactive oxygen species
SBD	Substrate binding domain
sGC	Soluble guanylyl cyclase
SHR	Spontaneously hypertensive rat
SR	Sarcoplasmic reticulum
T1D	Type 1 diabetes
T2D	Type 2 diabetes
TLR	Toll-like receptor
TLR4-MD2	Toll-like receptor 4 and myeloid differentiation factor 2 complex
TNF-α	Tumor necrosis factor alpha
VSMC	Vascular smooth muscle cells
